# The short-term effect of ambient particulate matter on suicide death

**DOI:** 10.1186/s12940-023-01042-2

**Published:** 2024-01-03

**Authors:** Tae-Hwa Go, Min-Hyuk Kim, Yoon-Young Choi, Jaehyun Han, Changsoo Kim, Dae Ryong Kang

**Affiliations:** 1 Department of Biostatistics, Yonsei University Wonju College of Medicine, Wonju, Republic of Korea; 2 Department of Psychiatry, Yonsei University Wonju College of Medicine, Wonju, Republic of Korea; 3 Artificial Intelligence BigData Medical Center, Yonsei University Wonju College of Medicine, Wonju, Republic of Korea; 4 Yonsei University Wonju College of Medicine, Wonju, Republic of Korea; 5 Department of Preventive Medicine, Yonsei University, Seoul, Republic of Korea; 6 Department of Precision Medicine and Biostatistics, Yonsei University Wonju College of Medicine, Wonju, Republic of Korea

**Keywords:** Particulate matter, Suicide, Time-series analysis

## Abstract

**Background:**

Air pollution can cause various respiratory and neurological diseases and continuous exposure can lead to death. Previous studies have reported that particulate matter (PM) exposure increases the risk of depression, suicidal thoughts, and suicidal death; however, the results are inconsistent and limited. This study aimed to examine the relationship between short-term PM exposure and suicide deaths, as well as investigate the short-term effects of PM on suicide death within vulnerable groups based on factors such as sex, age group, suicide-related information (note, method, and cause), psychiatric disorders, and physical diseases.

**Methods:**

Data on a total of 28,670 suicide deaths from 2013 to 2017, provided by the Korea Foundation for Suicide Prevention, were analyzed. The study design employed a time-series analysis with a two-stage approach. In the first step, a generalized additive model combined with a distributed lag nonlinear model was used to estimate the short-term effect of PM exposure on suicide risk specific to each city. In the second step, the estimated results from each city were pooled through a meta-analysis to derive the overall effect. We determined the effects of single lag, cumulative lag, and moving average PM concentrations from days 0–7 before suicide.

**Results:**

We confirmed an association between exposure to PM_10_ (≤ 10 μm in diameter) and deaths due to suicide. In particular, among individuals with psychiatric disorders and those who employed non-violent suicide methods, increased exposure to PM_10_ was associated with a higher risk of death by suicide, with percentage changes of 5.92 (95% confidence interval [CI]: 3.95–7.92) and 11.47 (95% CI: 7.95–15.11), respectively. Furthermore, in the group with psychiatric disorders, there was an observed tendency of increasing suicide risk as PM_10_ levels increased up to 120 µg/m^3^, whereas in the group with non-violent suicide deaths, there was a pronounced trend of rapid increase in suicide risk with an increase in PM_10_ up to 100 µg/m^3^.

**Conclusions:**

These results show an association between short-term exposure to PM and suicide. Our study adds evidence for the benefits of reducing PM in preventing diseases and improving mental health.

**Supplementary Information:**

The online version contains supplementary material available at 10.1186/s12940-023-01042-2.

## Background

Suicide is the leading cause of death worldwide, accounting for over 700,000 deaths annually [1]. In particular, Korea’s suicide mortality rate has been declining since 2011 but is still the highest among OECD countries [2]. Suicide being the number one cause of death for people in their 10s, 20s, and 30s in Korea [3] is a significant problem for society. However, risk factors for suicide require further understanding. Studies have identified several factors associated with suicide risk, including individual-level factors (history of depression or other mental disorders, experience of suicide attempts, physical characteristics, and demographic characteristics), psychological factors, biological factors (genetic, epigenetic, and neurobiological), and environmental factors [4–9]. These factors combined can have synergistic effects on suicidal behavior [10], and modifiable or potentially treatable risk factors are important considerations in research. Among these risk factors, air pollution, an environmental factor, is an important risk factor related to adverse health effects. Increased concentrations of air pollutants, particularly particulate matter (PM), have consistently been associated with increased respiratory and cardiovascular mortality and morbidity worldwide [11]. Recently, it has been hypothesized that air pollution can also induce self-aggressive behavior by increasing the levels of cytokines and reactive oxygen species [12], and that short- and long-term exposure to air pollution increases the risk of mental health-related diseases [13–15]. In addition, with increasing PM exposure, hospitalization and emergency room visits due to mental illness have increased [16, 17]. Furthermore, several studies have reported an association between PM exposure and suicide-related deaths [18–21], and the varying diameters of PM affect suicide risk differently [19, 20, 22]. The neurotoxic effects of air pollution can be particularly dangerous to vulnerable populations such as people with mental illnesses. In patients with major depressive disorder, a high-risk group for suicide, there was an association between short-term exposure to PM and suicide, and a clear dose-response relationship between short-term exposure to PM and suicide-related death was confirmed [23]. Furthermore, an association has been reported between long-term PM exposure and suicide in patients with physical or mental diseases, and a dose-response relationship has been confirmed [24]. Several studies have identified an association between PM and suicide stratified by sex and age group. Compared to men, the association between PM and suicide was stronger in women [16, 23]. Although no significant association in individuals aged 20–39 years was found, in those aged 40 years or older, the suicide risk tended to increase when both particulate matter ≤ 10 μm in diameter (PM_10_) and particulate matter ≤ 2.5 μm in diameter (PM_2.5_) concentrations increased [23]. However, these relationships vary across studies, and the evidence supporting the association between PM and suicide is limited. Considering the analysis in various stratified groups, it is necessary to identify the groups vulnerable to the effects of PM. Our research team conducted a study of the association between PM and suicide deaths in 2010, and we would like to report our latest findings. Therefore, this study aimed to: (1) investigate the relationship between short-term exposure to PM and suicide death and (2) investigate the short-term effects of PM on suicide in vulnerable groups based on factors including sex, age group, suicide note, psychiatric disorder, physical disease, main suicide method, and main suicide cause.

## Methods

### Data source and study population

The data on suicide deaths in South Korea from January 1, 2013 to December 31, 2017 were obtained from the Korea Foundation for Suicide Prevention. The data were collected by reviewing official police investigation records. The data included personal information about suicide deaths (such as resident registration number, age, and sex), location of discovery, and method and main cause [25].

In this study, complete enumeration data of suicide-related deaths were used. In these data, suicide death was defined as cases falling in the category of intentional self-harm (X60-X84) of the 10th Amendment of the International Classification of Diseases and Related Health Issues (ICD-10). The study population included suicide-related deaths in Seoul, Gyeonggi, and Incheon, metropolitan areas in South Korea. The total number of subjects was 28,670. For the analysis, the index date was defined as the date of suicide death or the date of discovery. If the date of death was earlier than the date of discovery, the index date was defined as the date of death; if the date of death was later than the date of discovery (died after discovery), the index date was defined as the date of discovery. The index place was the place of discovery and was divided into 66 cities and districts. The number of suicide deaths was calculated based on dates and cities/district, and it was used in conjunction with environmental data for analysis. This study was approved by the Institutional Review Board of Yonsei University Wonju College of Medicine, Republic of Korea (CR320301). Data were anonymized using a randomly generated number, and no personally identifiable information of the suicide was included in the study.

### Air pollution and meteorological data (environmental data)

The Korea Environment Corporation AirKorea provides hourly average concentration data of air pollution levels for sulfur dioxide (SO_2_), carbon monoxide (CO), nitrogen dioxide (NO_2_), ozone (O_3_), PM_10_, and PM_2.5_ collected by 614 measurement stations in 162 cities. We obtained SO_2_, CO, NO_2_, O_3_, and PM_10_ data for the period 2013–2017 measured at 160 measurement stations in Seoul, Gyeonggi, and Incheon, and PM_2.5_ data for the period 2015–2017. Air pollution data were provided as 1-h concentrations and converted into 24-h average concentrations for analysis. Meteorological data on temperature, humidity, sunlight, and air pressure at the city level, were obtained from the Korea Meteorological Administration. The data provided were in hourly units, but we converted them to 24-h average concentrations for analysis.

### Stratification variables

The stratification variables considered in the study were sex (men/women), age (19–40, 41–64, or > 65 years), suicide notes (presence or absence), psychiatric disorders (with or without), physical disease (with or without), main suicide method (violent or non-violent), and main suicide cause (economic, sociological, or health problem). Physical diseases were determined using statements and medical certificates provided by the bereaved family as documented in the police investigation records and claims data from the National Health Insurance Service. The presence or absence of psychiatric disorders was established by the data investigator. Consequently, if it was not assessed, it was not recorded, resulting in numerous missing values for this variable. The same applies to variables related to physical diseases. Methods of suicide were documented as the main suicide methods. When two or more suicide methods were used, they were recorded in consideration of being registered as the direct cause of death in the investigation record. If there were more than two direct causes of death, they were recorded based on criticality. Violent methods included hanging, jumping from a height, jumping in front of a car or train, cutting and piercing with sharp objects, using firearms or shotguns, and self-immolation. Non-violent methods included ingestion of pesticides, drug overdose, gas poisoning, and suffocation [26]. In addition, the main cause of suicide was recorded as the factor that caused severe pain to the person who committed suicide and had the strongest influence on suicide among the stress factors that lasted until death by reviewing the investigation records. The cause of suicide was classified into occupational problems, economic problems, family problems, interpersonal problems, physical health problems, mental health problems, and other causes. The other causes included academic, political, religious, and unknown problems. For our analysis, we categorized main suicide causes into two groups: socio-economic problems (combining occupational, economic, family, and interpersonal problems) and health problems (combining physical and mental health problems). Finally, as a warning sign, we considered any sign that indicated a suicidal individual’s thoughts or intentions regarding suicide, manifested through linguistic, behavioral, or emotional expressions prior to the suicide. Language signs included directly conveying words suggesting death to the people around the person, primarily family members, or frequently bringing up stories related to suicide. Behavioral signs included a loss of interest in appearance, self-harm behaviors, or cleaning up their surroundings, such as their bank account and home. Emotional signs encompassed changes in emotional states such as guilt, sadness, or emotional distress. While warning signs are closely related to suicide, they were not included as stratification variables and are only presented in Table 1 as baseline characteristics.

**Table 1:** Baseline characteristics of suicide death cases

Variables	*N* (%) *N* = 28,670
Region	
Seoul	9,923 (34.6)
Gyeonggi	14,986 (52.3)
Incheon	3,761 (13.1)
Sex (male)	19,732 (68.8)
Age	51.8 ± 17.8
Under 19 years	456 (1.6)
19–40 years	7,769 (27.1)
41–64 years	13,158 (45.9)
Over 65 years	7,287 (25.4)
Suicide method	
Violent method	21,970 (76.7)
Hanging	15,725 (54.9)
Drowning	637 (2.2)
Cutting and piercing	376 (1.3)
Jumping in front of a car or train	86 (0.3)
Others	106 (0.4)
Non-violent method	6,679 (23.3)
Drug poisoning	2,207 (7.7)
Gas poisoning and suffocation	4,472 (15.6)
Suicide cause, *N* = 27,585	
Occupational problems	1,322 (4.6)
Economic problems	6,007 (21.0)
Family problems	3,039 (10.6)
Interpersonal problems	1,404 (4.9)
Physical health problems	4,605 (16.1)
Mental health problems	10,548 (36.8)
Other causes	660 (2.3)
Suicide note (Presence)^†^, *N* = 26,336	11,150 (42.3)
Physical disease (With)^†^, *N* = 20,582	10,921 (53.1)
Psychiatric disorder (With)^†^, *N* = 16,461	8,780 (53.3)
Warning signal (language)^†^, *N* = 22,959	20,298 (88.4)
Warning signal (behavior)^†^, *N* = 20,521	15,372 (74.9)
Warning signal (emotion)^†^, *N* = 21,326	18,900 (88.6)

*Data are presented as number (percentage) or mean ± standard deviation

^†^These variables had missing values; *N* indicates the number of subjects included in the analysis

### Statistical analysis

The association between PM exposure and suicide was investigated using a time-series analysis design. A two-stage analysis was conducted in this study. The first step estimated the city-specific effect using a generalized additive model combined with a distributed lag nonlinear model to estimate the short-term effect of PM exposure on suicide risk. Daily suicide deaths were used as the dependent variable, PM was used as the independent variable, and a quasi-Poisson model was applied to adjust for overdispersion. Daily weather data such as temperature, relative humidity, air pressure, and sunlight, time trends, and holidays were considered as covariates. The time-series analysis model was as follows:

$$\begin{gathered}log\left( {E\left[ {{Y_t}} \right]} \right) = \alpha + \beta \left( {PM} \right) \hfill \\\,\,\,\,\,\,\,\,\,\,\,\,\,\,\,\,\, + ns\left( {temperature,df = 6} \right)\, + ns\left( {relative\,humidity,df = 3} \right) \hfill \\\,\,\,\,\,\,\,\,\,\,\,\,\,\,\, + ns\left( {air\,pressure,df = 3} \right)\, + ns\left( {sunlight,df = 3} \right) \hfill \\\,\,\,\,\,\,\,\,\,\,\,\,\, + ns\left( {calendar\,time,df = 7} \right) + holiday \hfill \\ \end{gathered}$$

where $E\left({Y}_{t}\right)$ is the number of suicide deaths on day $t$, $\alpha$ is the model intercept, $\beta$ indicates the log-relative risk of suicide associated with an interquartile range (IQR) increase in the PM level, and $ns\left( \right)$ represents the natural cubic spline function. To remove the seasonal trend of time-series data, seven degrees of freedom per year were used for time, the smooth function of a natural cubic spline with six degrees of freedom was used for temperature, and the smooth function of a natural cubic spline with three degrees of freedom was used for relative humidity, sunlight, and air pressure. The IQR was calculated as the difference between the third and first quartile each for PM_10_ and PM_2.5_ during the study period. In the second stage, a meta-analysis was performed using a fixed and random effects model after confirming heterogeneity to estimate the overall effect using the estimated values from the city-specific model in the first stage.

The effect of PM exposure on suicide was evaluated as a single lag and a moving average lag effect. The single lag effect confirmed the air pollution exposure effect from the day of suicide to 7 days before the day of suicide, and the moving average effect was estimated as the moving average up to 1, 3, 5, and 7 days prior to the day of suicide. The effect was expressed as the percentage change (95% confidence interval [CI]) in the association between the increased IQR of PM and the risk of suicide.

Additionally, subgroup analysis was conducted for stratification variables using a moving average lag 03 model. The percent change (95% CI) in suicide risk was assessed for each IQR increase in PM. Furthermore, the exposure-response curves were plotted to confirm the association between PM exposure and suicide risk [27]. To compare relative risks between subgroups, we conducted Z-tests. For sensitivity analysis, the concentration of air pollutants other than PM was adjusted, and the association between PM and suicide-related deaths was confirmed through a two-pollution model. In addition, because PM and suicide data exhibit seasonality, a seasonal stratification analysis was conducted. All analyses were reported based on the pooled estimates from a meta-analysis, combining data from 66 cities and districts. A two-sided P-value < 0.05 was considered statistically significant and the analyses were performed using SAS version 9.4 (SAS Institute Inc., Cary, NC, USA), R studio, and R version 4.2.1 (R Foundation for Statistical Computing, Vienna, Austria).

## Results

### Baseline characteristics

Table 1 shows the general characteristics of the suicide-related deaths. From 2013 to 2017, the number of suicide-related deaths in Seoul, Gyeonggi, and Incheon was 28,670, with an average age of 52 years; 69% of the suicide victims were male. Violent methods accounted for 77% of the suicide methods, and mental health and economic problems were the most common causes of suicide. Most deaths due to suicide had prior warning signs.

### Air pollution and meteorological data

The distributions of the average PM concentrations and meteorological data during the study period are shown in Table 2. The average concentrations were 50.736 µg/m³ for PM10 and 26.011 µg/m³ for PM2.5, with IQRs of 29.12 µg/m³ and 16.68 µg/m³, respectively. The average concentrations of other air pollutants were 0.005 ppm for SO_2_, 0.552 ppm for CO, 0.024 ppm for O_3_, and 0.030 ppm for NO_2_ (Supplementary Table 1, see Additional file 1). The daily average temperature was 12.9 ℃, the relative humidity was 66%, the duration of sunshine was 0.51 h, and the air pressure was 1009.89 hPa. Supplementary Table 2 (see Additional file 1) shows the correlations between air pollution and meteorological data. The relationships differed between substances, but there were significant positive correlations between each air pollutant. The correlation between PM_10_ and PM_2.5_ was the highest.

**Table 2:** Distribution of PM concentrations and meteorological factors in three Korean cities from 2013 to 2017

Variables	Mean	SD	Min	Percentile			Max
				25	50	75	
Air pollutants							
PM_10_ (µg/m^3^)	50.736	28.318	2.125	33.065	45.482	62.182	604.83
PM_2.5_ (µg/m^3^)^a^	26.011	14.147	0.042	16.236	23.377	32.917	143.333
Weather conditions							
Temperature (℃)	12.9	10.5	-16.7	3.4	14.2	22.4	31.4
Relative humidity (%)	66.0	15.3	15.8	55.13	66.38	76.78	100.0
Sunshine (hr)	0.51	0.30	0.00	0.25	0.59	0.78	0.96
Air pressure (hPa)	1009.89	8.81	980.36	1002.96	1009.92	1016.39	1035.71

PM_10_, particulate matter ≤ 10$\mu m$ in diameter; PM_2.5_, particulate matter ≤ 2.5$\mu m$ in diameter; SD, Standard deviation

^a^ PM_2.5_ only consisted of data from 2015 to 2017

### Association between suicide-related death and PM

Table 3 shows the association between PM exposure and suicide among all the subjects. The results confirmed that the suicide risk increased by 2.9% when the IQR of PM_10_ in lag increased, but there were no significant associations at other single lags. An association was found between increased PM_10_ and suicide risk in all moving averages. However, there was no statistically significant association between suicide and PM_2.5_.

**Table 3:** Association between the risk of suicide death and PM per IQR increase

Lag	PM_10_	PM_2.5_
	Percentage change (95% CI)	Percentage change (95% CI)
Single lag		
Lag 0	2.91 (0.57–5.30)	0.88 (-2.54–4.42)
Lag 1	0.32 (-1.69–2.36)	-1.03 (-4.96–3.06)
Lag 2	0.77 (-1.49–3.09)	-0.84 (-5.01–3.51)
Lag 3	0.88 (-1.13–2.93)	2.81 (-1.26–7.03)
Lag 4	0.58 (-1.40–2.61)	0.51 (-4.23–5.48)
Lag 5	1.73 (-0.34–3.84)	1.73 (-2.32–5.94)
Lag 6	-1.34 (-3.61–0.99)	-3.44 (-7.25–0.52)
Lag 7	1.81 (-0.16–3.82)	1.61 (-1.75–5.08)
Moving average		
Lag 01	4.85 (3.44–6.28)	0.67 (-1.91–3.33)
Lag 03	6.21 (4.45–8.01)	1.24 (-1.89–4.46)
Lag 05	7.75 (5.55–9.99)	1.36 (-2.26–5.10)
Lag 07	8.75 (6.00–11.56)	0.65 (-3.36–4.84)

*PM_10_, particulate matter ≤ 10$\mu m$ in diameter; PM_2.5_, particulate matter ≤ 2.5$\mu m$ in diameter

The IQR was 29.12 µg/m^3^ for PM_10_ and 16.68 µg/m^3^ for PM_2.5_. The model included temperature, relative humidity, air pressure, sunlight, calendar time, and holidays as adjustment variables

IQR, interquartile range; PM, particulate matter

**Fig. 1:**
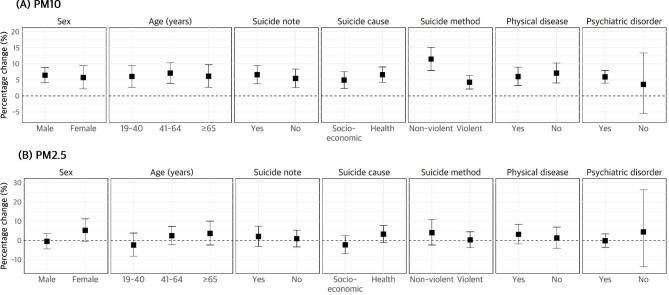
Association between the risk of suicide death and PM (lag 03) per IQR increase by stratified subgroup. *Data are presented as percentage changes (%) with 95% confidence intervals. The model included temperature, relative humidity, air pressure, sunlight, calendar time, and holidays as adjustment variables. IQR, interquartile range; PM, particulate matter

**Fig. 2:**
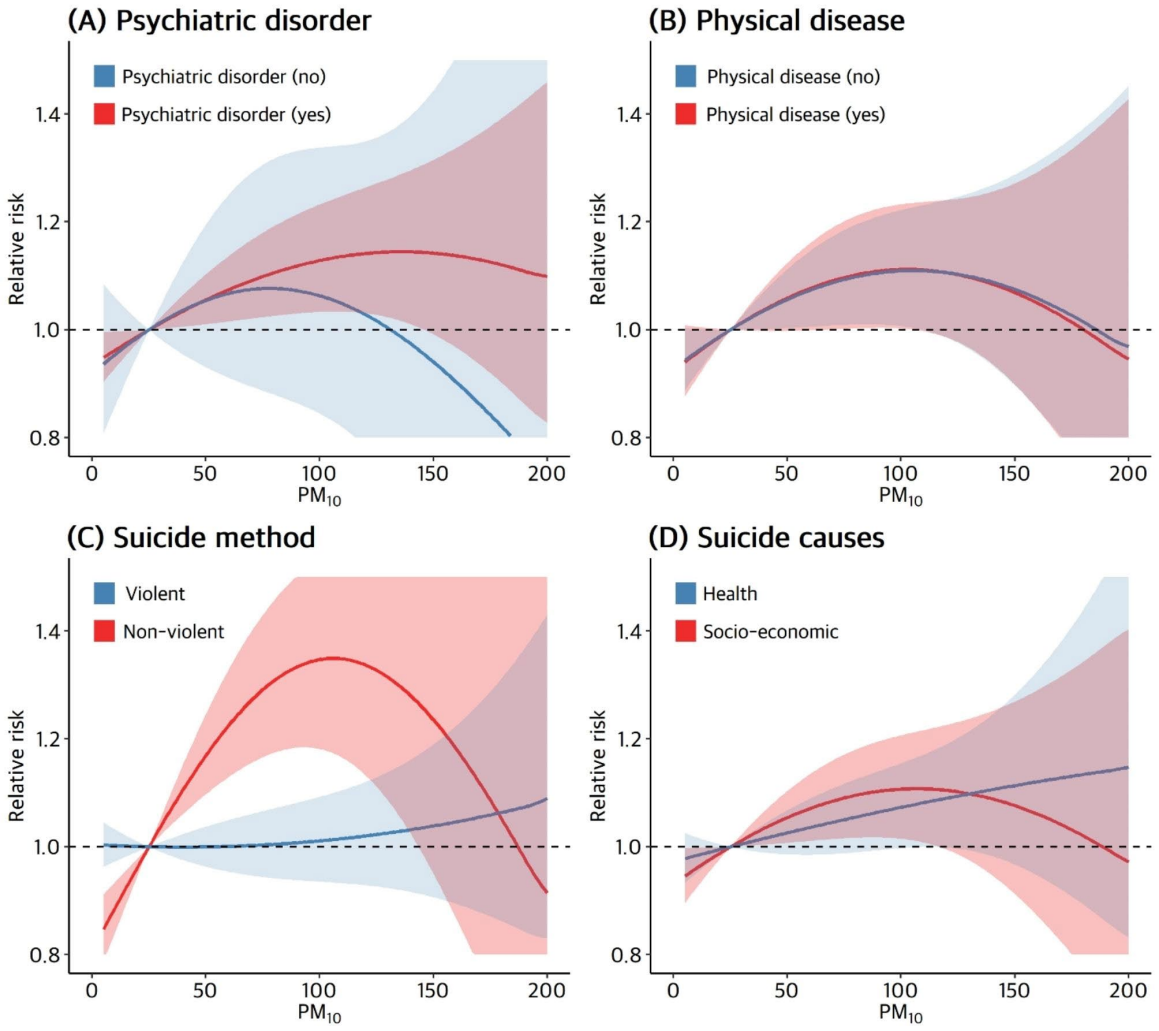
Exposure-response curve for the association between risk of suicide death and PM_10_ (lag 03) by stratified subgroup. *Data are presented as relative risk with 95% confidence intervals. The model included temperature, relative humidity, air pressure, sunlight, calendar time, and holidays as adjustment variables. PM, particulate matter

Figure 1 shows the association between PM exposure and suicide in stratified subgroups (lag 03). The association between PM_10_ and suicide was statistically significant in almost all the subgroups. The risk of suicide increased by 6.45% (95% CI: 4.15–8.81) in males and 5.74% (95% CI: 2.17–9.43) in females, as well as by 6.07% (95% CI: 2.71–9.55) in those aged 19–40 years, 7.10% (95% CI: 3.94–10.35) in those aged 41–64 years, and 6.16% (95% CI: 2.72–9.72) in those aged over 65 years. Regarding factors related to suicide, the risk ratio was similar regardless of the presence or absence of a suicide note, and the risk of suicide stratified by the main suicide cause was 6.62% (95% CI: 4.29–9.00) for health problems and 4.92% (95% CI: 2.32–7.58) for socio-economic problems. Although the risk of suicide was higher for the group with health problems than for that with socio-economic problems, this difference was not statistically significant. Depending on the suicide method, the association between PM_10_ and suicide deaths was stronger for suicides using non-violent methods (4.25%, 95% CI: 2.15–6.40) than for suicides using violent methods (11.47%, 95% CI: 7.95–15.11). Notably, this was the only subgroup analysis with a significant difference (*P* = 0.001). Additionally, the risk of suicide was similar according to the presence or absence of physical problems. In the absence of psychiatric disorders, the association between PM_10_ and suicide deaths was not statistically significant, whereas the risk of suicide increased by 5.92% (95% CI: 3.95–7.92) if psychiatric disorders were present. However, the risk was not significantly different between these two groups. In contrast, for PM_2.5_, the association with suicide risk was not statistically significant. In Fig. 2, the association between PM_10_ (lag 03) and the risk of suicide in each subgroup is shown as an exposure–response curve. In the group with mental disorders, the risk of suicide tended to increase with an increase in PM_10_ up to 120 µg/m³, and in the non-violent suicide-related death group, the risk of suicide increased rapidly with an increase in PM_10_ up to 10 µg/m³.

### Sensitivity analysis

After adjusting for air pollutants other than PM, the association between moving average day 3 of PM and suicide-related deaths was confirmed, as shown in Table 4. After adjusting for other air pollutants, we confirmed a statistically significant association between increased PM_10_ and suicide. After adjusting for CO, which had the highest correlation with PM_10_, an increase in the IQR of PM_10_ increased the suicide risk by 9.28% (95% CI: 6.17–12.50). Even after adjusting for SO_2_, O_3_, and NO_2_, the risk of suicide increased by 6.16% (95% CI: 3.60–8.78), 6.58% (95% CI: 4.09–9.14), and 7.32% (95% CI: 4.80–9.91), respectively. In contrast, even after adjusting for other air pollutants, there was no statistically significant association between PM_2.5_ and suicide risk.

**Table 4:** Association between the risk of suicide death and PM (lag 03) per IQR increase controlled for other air pollutants

PM	Adjusted air pollution	Percentage change (95% CI)
PM_10_	SO_2_	6.16 (3.60–8.78)
	CO	9.28 (6.17–12.50)
	O_3_	6.58 (4.09–9.14)
	NO_2_	7.32 (4.80–9.91)
PM_2.5_	SO_2_	2.24 (-1.92–6.57)
	CO	2.76 (-2.40–8.20)
	O_3_	1.12 (-2.51–4.89)
	NO_2_	-0.81 (-5.25–3.84)

SO_2_, sulfur dioxide; CO, carbon monoxide; NO_2_, nitrogen dioxide; O_3_, ozone; PM_10_, particulate matter ≤ 10 μm in diameter; PM_2.5_, particulate matter ≤ 2.5 μm in diameter. The model included temperature, relative humidity, air pressure, sunlight, calendar time, and holidays as adjustment variables. IQR, interquartile range; PM, particulate matter

Seasonal stratification analysis results are presented in Supplementary Table 3 (see Additional file 1). In spring, when PM exposure was the highest, the association between increased PM_10_ and suicide risk was the greatest, but there was no association between increased PM_2.5_ and suicide risk.

## Discussion

This study investigated the association between short-term exposure to PM and suicide-related deaths in 28,670 people who died by suicide in large cities in Korea. The analysis results showed that the risk of suicide-related death increased with short-term exposure to PM_10_, and this short-term effect of PM_10_ was consistent and robust even after adjusting for other air pollutants. Subgroup analysis confirmed an association between PM_10_ exposure and suicide in all groups except subjects without psychiatric disorders. However, no association was found between exposure to PM_2.5_ and suicide.

Several studies have reported an association between short-term PM exposure and suicide. In a study similar to our study, that examined the association between air pollution and suicide deaths in 10 cities in Northeast Asia, the association between PM_10_ and suicide death was confirmed, while PM_2.5_ was not associated with suicide death [20]. Lin et al. reported an association between short-term exposure to PM_10_ and suicide in a group of suicide victims in Guangzhou, China [21]. In addition, in a previous study conducted by this research team in 2010, it was reported that the risk of suicide increased by 9.0% and 10.1% when the IQR of PM_10_ and PM_2.5_ increased, respectively [18]. However, several studies have reported results that contradict our findings [17, 19, 22].

It is well known that exposure to air pollution can activate cytokines that trigger the body’s inflammatory response, thus increasing the systemic inflammatory response and oxidative stress [28, 29]. Experimental studies have shown that long-term exposure to air pollution can lead to neuroinflammation and oxidative stress, leading to brain pathologies that increase the risk of developing depression [30, 31]. In addition, heavy metals, such as lead and mercury, contained in PM can affect the central nervous system and cause depression or suicidal thoughts. Thus, short-term changes in air pollution can cause significant changes in health status based on changes previously induced by long-term exposure to air pollution. Suicide can be influenced by a combination of factors and requires risk management in vulnerable populations.

Subgroup analyses were conducted in stratified groups by sex, age group, suicide notes, psychiatric disorders, physical disease, main suicide method, and main suicide cause and compared the risk of PM and suicide within subgroups. As a result, statistically significant difference was found only in the main suicide method, indicating that individuals who attempt suicide using non-violent methods are more affected by PM_10_ exposure compared to those who attempt suicide through violent methods. However, no significant differences were observed in other subgroups. In contrast to our findings, Lin et al. reported that PM exposure affects suicide risk only in groups that attempted suicide by violent methods, based on the fact that seasonal changes were significantly correlated with violent suicide-related deaths but not with non-violent suicide-related deaths [21, 32]. However, Sun et al. reported the presence of seasonality among non-violent suicide cases [33]. A psychological autopsy study comparing violent and non-violent suicides among elderly individuals in rural China found that non-violent suicide cases had higher levels of depression compared to violent suicide cases and emphasized that depression and hopelessness were independent risk factors for non-violent suicide [26]. Considering that depression and hopelessness are risk factors for suicidal ideation and death [34], it is possible that non-violent suicide cases may be more vulnerable to environmental factors such as PM.

PM_10_ and suicide-related deaths were not associated in individuals without psychiatric disorders, but our data confirmed an increased risk of suicide in individuals with psychiatric disorders when the IQR of PM_10_ was increased. Patients who visited the hospital for depression may have had preexisting depressive symptoms, and short-term changes in air pollution may trigger severe depressive symptoms in patients with prior moderate disease manifestation. As PM is expected to affect suicide-related deaths more sensitively in patients with mental illnesses, it is necessary to recommend the prevention of PM exposure in such patients.

Similar to our study, Kim et al. [20] reported no clear pattern in effect modification by sex and age. However, some studies [18, 19] have shown that PM has a stronger impact on suicide in men, while other studies [23, 35] have reported a more significant effect on women. For men, PM exposure may increase due to increased outdoor activities, and for women, PM exposure may be related to the inflammatory effects of estrogen and the immunosuppressive effect of androgens [23]. The effects of PM exposure according to age groups were reported diversely, and the range of age groups was extensive [18, 21, 23, 36–38]. In the study by Casas et al. [39], which tested for effect modification, the effect of PM_10_ on suicide was only confirmed in extreme age groups - children (5–14 years) and the elderly (over 85 years), showing significant p for interaction. However, in our study, a subgroup analysis for patients under 18 years was not conducted due to a small number of cases, so the results for extreme age could not be confirmed. Lin et al. [21] have suggested that younger people are more likely to work outdoors, increasing their exposure to air pollution. Since our study focused on the metropolitan area, there might have been less variation in occupational differences between age groups compared to other regions, which could have resulted in no differences in the PM effect across age groups.

Furthermore, we have not found suicide causes, physical disease, and suicide note as effect modifiers. Individuals with socio-economic problems may have an increased risk of suicide due to the stress and psychological pressure resulting from these issues and may not have received appropriate treatment for mental health problems or depression due to economic problems. People with physical problems may experience inherent stress and depression, and those already suffering from health problems could be more vulnerable to environmental factors. In addition, previous studies [18, 23] have reported a PM effect on suicide among subjects with physical diseases, with most of these studies assessing the impact based on the type of illness (e.g., cardiovascular disease, cancer, depression, etc.). However, we analyzed using only the presence or absence of physical illnesses, which might explain why the differences were not as clear. Considering factors such as the type of illness, its severity, and management status would be necessary to establish a clear relationship. There are very few studies that have examined the impact of PM exposure by the presence or absence of a suicide note. In studies comparing people who wrote suicide notes to those who didn’t, it was reported that those who left notes often had issues like intimate partner problems, as well as financial difficulties or illnesses [40]. It is speculated that the relatively minor impact of PM exposure may not have been pronounced due to the complex interplay of various factors that can already influence the risk of suicide.

The strength of this study is that it included subgroups based on various suicide-related factors. The results of the subgroup analysis can provide insights into vulnerable populations and have implications for research and public health management. As the data used in this study were collected based on official police investigation records of suicide victims, it was possible to investigate data closely related to suicide. In addition, we were able to study the relationship between PM exposure and suicide-related death using information on the cause of the suicide and through interviews with bereaved families and in-depth analysis of the progression of suicidal behavior. These research findings can serve as a basis for identifying vulnerable populations and developing suicide prevention strategies within various subgroups by examining the relationship between PM and suicide. Furthermore, we analyzed the concentration of PM based on the smallest unit area centered on metropolitan areas. According to data from the Korea Psychological Autopsy Center, there is a large difference in the number of suicide-related deaths and the average suicide rate by city, county, and district in Korea [25]. In addition, even if the physical distance is small, the PM concentration varies greatly from region to region owing to differences in geographic characteristics. Air pollution monitoring stations are limited, and their analyses may not be sufficient for analyzing the long-term effects of PM, especially due to changes in administrative districts and monitoring stations in the past. Therefore, we conducted a study based on metropolitan areas where PM was consistently measured and used PM exposure information by region divided into fine units. In particular, we attempted to quantify PM exposure by considering each individual’s actual residence and suicide attempt area. Finally, a time-series analysis design was used in this study. We expected that a time-series analysis design, rather than a case-crossover analysis design, would provide more reliable outcomes because the residuals have less autocorrelation [41, 42].

Our study had several limitations. First, suicide cases may have been underestimated because only those who died after a suicide attempt were included, and not those who did not die. Second, in Korea, PM_2.5_ data have been recorded since 2015; therefore, only data from a total of 3 years could be used, which may not include enough cases. Third, the number of deaths by suicide was low compared with other disease studies. This may be the reason for the lack of a significant association in the sub-analysis. In previous studies investigating the relationship between PM and mental health outcomes, researchers have included data spanning more than 5 years for mental health-related outcomes, aiming to overcome these limitations [17, 19, 35, 43]. The limited number of cases may result in a lower level of confidence in the research findings. However, the low incidence of suicide deaths is an unavoidable restriction of our study. Fourth, a subgroup analysis of patients younger than 18 years of age was not performed because of the small number of cases. As the brain and respiratory system are still developing in children and teenagers, they are considered more vulnerable to air pollution, and future studies should focus on individuals in this age group [16]. Fifth, various variables considered in this study have a relatively high proportion of missing data. Particularly, data on physical and mental diseases are missing in 28.2% and 42.6% of cases, respectively. Despite the potential for decreased reliability, the presence of physical illnesses and psychiatric disorders is crucial for assessing suicide risk. Therefore, these factors could not be excluded from the analysis, as they play a vital role in understanding the risk. However, for a generalization of our results, further extensive research with a larger dataset is required. Finally, only the presence or absence of mental diseases was considered, although the aggravation of mental diseases is known to increase the risk of suicide, and there are many stages of mental diseases. To evaluate the increased vulnerability due to PM exposure, it will be necessary to specifically evaluate the impact on mental health, as well as physical diseases.

It is difficult to define the characteristics of a suicidal person because suicide can be induced by a combination of various factors. Although there are known important risk factors, the complexity increases when combined with various individual characteristics; furthermore, various types of suicide exist, making it difficult to isolate the contribution of PM exposure. Performing stratification analysis in environmental epidemiology allows us to analyze these complex factors separately.

In conclusion, we investigated the effects of PM exposure on suicide-related deaths using stratified analysis. Previous studies have demonstrated that air pollution can increase the risk of mental disorders. Our research findings also show an association between PM_10_ and suicide, and we have confirmed that this impact varies across subgroups. It suggests that PM needs to be carefully managed in vulnerable groups, and as most suicide victims show warning signs before death, sufficient management is considered essential for high-risk groups. Despite several limitations, we elucidated the association between PM and suicide deaths in various subgroups. We hope that our findings can serve as compelling evidence for disease prevention and improvements in mental well-being through the reduction of air pollution.

### Electronic supplementary material

Below is the link to the electronic supplementary material.

Additional file 1: **Fig. 1.** PM_10_, PM_2.5_ concentrations and Trend in Suicide Death in South Korea from 2013 to 2017. **Table (1)** Distributions of SO_2_, CO, O_3_, and NO_2_ concentrations in three Korean cities from 2013 to 2017. **Table (2)** Spearman’s coefficient of meteorological factors and air pollutants. **Table (3)** Association between the risk of suicide death and PM (lag 03) per interquartile range (IQR) increase by season.

## Data Availability

The data are not publicly available but will be provided upon request with the permission of the Korea Foundation for Suicide Prevention.

## List of Abbreviations

SO_2_: Sulfur dioxide
CO: Carbon monoxide
O_3_: Ozone
NO_2_: Nitrogen dioxide
PM: Particulate matter
PM_10_: Particulate matter ≤ 10 μm in diameter
PM_2.5,_: Particulate matter ≤ 2.5 μm in diameter
IQR: Interquartile range
CI: Confidence interval
