# Supplementary material for: The short-term effect of ambient particulate matter on suicide death
Source: Environ Health. 2024 Jan 3;23:3. doi: 10.1186/s12940-023-01042-2 (PMC10763266; doi:10.1186/s12940-023-01042-2)
Supplement: Supplementary file 1 — Additional file 1: Fig. 1. PM10, PM2.5 concentrations and Trend in Suicide Death in South Korea from 2013 to 2017. Table (1) Distributions of SO2, CO, O3, and NO2 concentrations in three Korean cities from 2013 to 2017. Table (2) Spearman’s coefficient of meteorological factors and air pollutants. Table (3) Association between the risk of suicide death and PM (lag 03) per interquartile range (IQR) increase by season. [file 12940_2023_1042_MOESM1_ESM.docx]

**Supplementary Fig. 1** PM_10_, PM_2.5_ concentrations, and Trend in Suicide Death in South Korea from 2013 to 2017


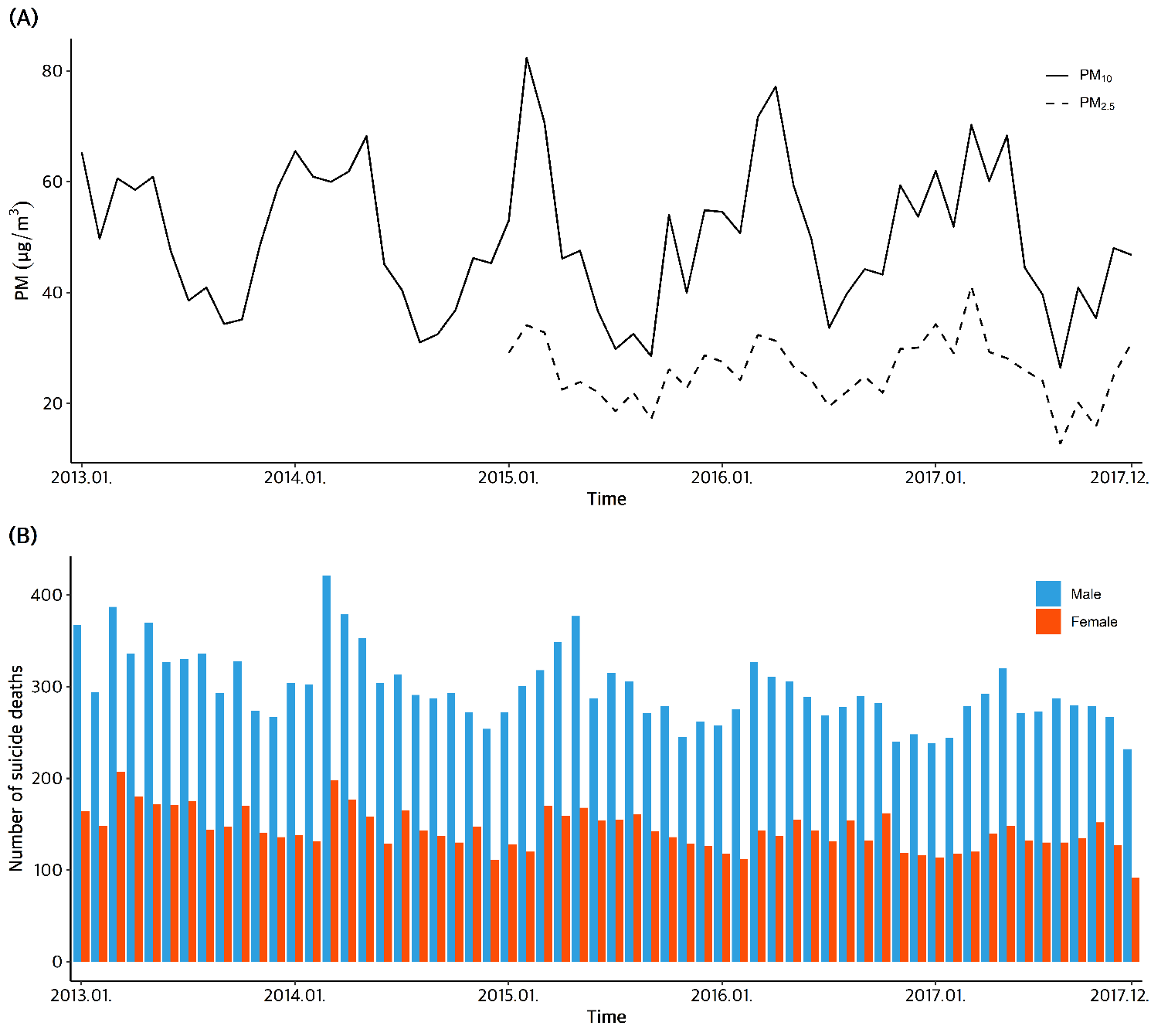
 *PM_2.5_ only consisted of data from 2015 to 2017.

**Supplementary Table. 1** Distributions of SO_2_, CO, O_3_, and NO_2_ concentrations in three Korean cities from 2013 to 2017

| Variables | Mean | SD | Min | Percentile | | | Max |
| --- | --- | --- | --- | --- | --- | --- | --- |
|  |  |  |  | 25 | 50 | 75 |  |
| Air pollutants |  |  |  |  |  |  |  |
| SO_2_ (ppm) | 0.005 | 0.002 | 0.001 | 0.004 | 0.004 | 0.006 | 0.021 |
| CO (ppm) | 0.552 | 0.202 | 0.100 | 0.414 | 0.512 | 0.642 | 2.135 |
| O_3_ (ppm) | 0.024 | 0.012 | 0.001 | 0.014 | 0.023 | 0.032 | 0.098 |
| NO_2_ (ppm) | 0.030 | 0.013 | 0.001 | 0.020 | 0.029 | 0.039 | 0.100 |

SO_2_, sulfur dioxide; CO, carbon monoxide; O_3_, ozone; NO_2_, nitrogen dioxide.

**Supplementary Table 2.** Spearman’s coefficient of meteorological factors and air pollutants

|  | SO_2_ | CO | O_3_ | NO_2_ | PM_10_ | PM_2.5_ | Temperature | Relative humidity | Sunshine | Air pressure |
| --- | --- | --- | --- | --- | --- | --- | --- | --- | --- | --- |
| SO_2_ | 1.000 |  |  |  |  |  |  |  |  |  |
| CO | 0.663^**^ | 1.000 |  |  |  |  |  |  |  |  |
| O_3_ | -0.090^**^ | -0.391^**^ | 1.000 |  |  |  |  |  |  |  |
| NO_2_ | 0.488^**^ | 0.710^**^ | -0.443^**^ | 1.000 |  |  |  |  |  |  |
| PM_10_ | 0.602^**^ | 0.693^**^ | 0.062^**^ | 0.511^**^ | 1.000 |  |  |  |  |  |
| PM_2.5_ | 0.596^**^ | 0.759^**^ | -0.010 | 0.550^**^ | 0.882^**^ | 1.000 |  |  |  |  |
| Temperature | -0.428^**^ | -0.495^**^ | 0.488^**^ | -0.293^**^ | -0.257^**^ | -0.186^**^ | 1.000 |  |  |  |
| Relative humidity | -0.142^**^ | -0.012 | 0.050^**^ | -0.242^**^ | -0.093^**^ | 0.114^**^ | 0.413^**^ | 1.000 |  |  |
| Sunshine | 0.118^**^ | -0.076^**^ | 0.241^**^ | -0.026^*^ | 0.021 | -0.083^**^ | -0.132^**^ | -0.535^**^ | 1.000 |  |
| Air pressure | 0.367^**^ | 0.461^**^ | -0.484^**^ | 0.336^**^ | 0.200^**^ | 0.206^**^ | -0.730^**^ | -0.339^**^ | 0.191^**^ | 1.000 |

SO_2_, sulfur dioxide; CO, carbon monoxide; O_3_, ozone; NO_2_, nitrogen dioxide; PM_10_, particulate matter≤10μm in diameter; PM_2.5_, particulate matter≤2.5μm in diameter

*p<0.05. **p<0.01.

**Supplementary Table 3.** Association between the risk of suicide death and PM (lag 03) per interquartile range (IQR) increase by season

| PM | Season | Percentage change  (95% CI) |
| --- | --- | --- |
| PM_10_ | Spring | 11.47 (7.95 – 15.11) |
|  | Summer | 4.25 (2.32 – 7.58) |
|  | Fall | 4.92 (2.32 – 7.58) |
|  | Winter | 6.62 (4.29 – 9.00) |
| PM_2.5_ | Spring | 4.02 (-2.36 – 10.81) |
|  | Summer | 0.28 (-3.77 – 4.49) |
|  | Fall | -2.29 (-6.83 – 2.48) |
|  | Winter | 3.27 (-1.05 – 7.77) |

PM_10_, particulate matter≤10μm in diameter; PM_2.5_, particulate matter≤2.5μm in diameter. The model included temperature, relative humidity, air pressure, sunlight, calendar time, and holiday as adjustment variables. IQR, interquartile range; PM, particulate matter.
